# The mitochondrial protein YME1 Like 1 is important for non-small cell lung cancer cell growth

**DOI:** 10.7150/ijbs.82217

**Published:** 2023-03-21

**Authors:** Yingchen Xia, Chunyan He, Zhi Hu, Zhichao Wu, Yin Hui, Yuan-yuan Liu, Chuanyong Mu, Jianhua Zha

**Affiliations:** 1 Department of Thoracic Surgery, The First Affiliated Hospital of University of Science and Technology of China (USTC), Division of Life Sciences and Medicine, University of Science and Technology of China, Hefei, China.; 2 Department of Clinical Laboratory, Kunshan Hospital of Chinese Medicine, Affiliated Hospital of Yangzhou University, Kunshan, China.; 3 Department of Thoracic Surgery, The First Affiliated Hospital of Nanchang University, Nanchang, China.; 4 Department of Thoracic Surgery, Changshu Hospital Affiliated to Nanjing University of Chinese Medicine, Changshu, China.; 5 Department of Thoracic Surgery, The First Affiliated Hospital of Shaoyang University, Shaoyang, China.; 6 Department of Thoracic Surgery, The Central Hospital of Shaoyang, Shaoyang, China.; 7 Department of Radiotherapy and Oncology, Affiliated Kunshan Hospital of Jiangsu University, Kunshan, China.; 8 Department of Respiratory and Critical Care Medicine, First Affiliated Hospital of Soochow University, Suzhou, China.; 9 Department of Thoracic Surgery, The First Affiliated Hospital of Nanchang University, Nanchang, China.

## Abstract

The expression and biological function of the mitochondrial inner membrane protease YME1L (YME1 Like 1 ATPase) in NSCLC are tested here. Bioinformatical analyses and results from local human tissues show that YME1L expression is elevated in NSCLC tissues. YME1L upregulation was observed in primary and immortalized NSCLC cells. In NSCLC cells, shRNA-mediated silence of YME1L or dCas9/sgRNA-induced knockout (KO) of YME1L robustly suppressed cell growth and migration, and provoking apoptosis. YME1L shRNA/KO resulted in mitochondrial dysfunctions in NSCLC cells, leading to mitochondrial depolarization, ROS accumulation and ATP depletion. Conversely, ectopic YME1L overexpression augmented NSCLC cell proliferation and motility. Akt-S6K1 phosphorylation was reduced after YME1L shRNA/KO in primary NSCLC cells, but augmented after YME1L overexpression. Importantly, YME1L KO-caused anti-NSCLC cell activity was attenuated by a constitutively-activate Akt1 (S473D) construct. *In vivo*, subcutaneous NSCLC xenograft growth was remarkably slowed following intratumoral YME1L shRNA AAV injection in nude mice. YME1L knockdown, Akt-mTOR inactivation and ATP reduction were detected in YME1L-silenced NSCLC xenografts. Taken together, overexpressed YME1L in NSCLC exerts pro-tumorigenic function.

## Introduction

Over 13% of all new cancers are lung cancer 1, 2. The most common non-small cell lung cancer (NSCLC) is one primary cause of global cancer mortality 3-5. The advanced NSCLC has high metastatic potential and malignancy 3-5. The present treatments, including surgical cancer resection, radiation, molecularly-targeted agents, immunotherapy (including PD1/PD-L1 blockers) and chemotherapies, have been failed significantly improve the prognosis of advanced NSCLC patients 3, 5.

The understanding of genetic alterations driving NSCLC is evolving in recent years 6-10. For example, mutations of EGFR, BRAF and MET as well as translocations of ALK, RET and NTRK are currently incorporated in the diagnostic standards of NSCLC 4, 5. Inhibitors and antibodies of these molecular targets have displayed promising efficiency in NSCLC patients, either alone or in combination of current chemotherapies 6-10. Novel targeted therapies of NSCLC are being explored 8, 11-13.

Mitochondria are vital for oxidative phosphorylation (OXPHOS), ATP production and amino acid metabolism, as well as macromolecules biosynthesis, fatty acid oxidation and ion homeostasis 14-18. Mitochondria act as the key hub for signaling transduction and apoptosis regulation 14-18. Mitochondrial functions are altered in NSCLC and other human cancers. In particular, increased bioenergetics in the rapidly proliferating tumor cells can meet their energy demand by generating more ATP 14-18. Increased mitochondrial respiration and ATP generation are vital for NSCLC tumorigenesis and progression 6, 19. For example, enhanced synthesis and/or uptake of heme will fuel elevated OXPHOS in NSCLC. Whereas suppressing uptake/synthesis of heme inhibited mitochondrial OXPHOS and robustly reduced oxygen consumption, thereby suppressing NSCLC growth 6, 19. Studies are focusing on understanding the mechanisms of mitochondrial alterations that are vital for tumorigenesis and development NSCLC 6, 14-19.

The mitochondrial protein YME1L (YME1 Like 1 ATPase) locates primarily at the inner mitochondrial (IM) membrane 20-24 and is important for maintaining mitochondrial morphology, functions and plasticity 22, 25, 26. Studies revealed that YME1L can regulate the degradation of different mitochondrial proteins 20-22, 27. Stiburek *et al.,* showed that YME1L knockdown in HEK293 cells impaired cell proliferation and changed cristae morphology, while inducing oxidative injury and decreasing rotenone-sensitive respiration 23. Silence of YME1L also induced accumulation of Ndufb6, ND1, Cox4 and other non-assembled respiratory chain subunits 23. In the present study the expression and possible biological functions of YME1L in NSCLC were studied.

## Methods

### Reagents

All cell culture reagents were provided by Hyclone (Logan, UT, USA). All chemicals were obtained from Sigma-Aldrich Co. (St. Louis, Mo, USA). The YME1L antibody was provided by Dr. Cao 28, 29. All other antibodies were reported previously 30, 31 or from Dr. Xu 32. Fluorescence dyes, including JC-1, DAPI, EdU and TUNEL as well as CellROX, Hoechst 33342were all from Thermo-Fisher Invitrogen (Carlsbad, CA, USA). The Histone-bound DNA ELISA Kit was purchased from Roche Diagnostic (Indianapolis, IN, USA). The “Transwell” chambers were provided by Corning Co. (New York, NY, USA).

### Cells

A549 cells were reported previously 30, 31 and were cultivated under RPMI medium plus serum. The primary NSCLC cells derived from three written-informed consent patients (pNSCLC-1, pNSCLC-2 and pNSCLC-3), the primary lung epithelial cells-derived from two written-informed consent donors were described early 30, 31, and cells cultured in medium described 30, 31. The Ethics Committee of Nanchang University approved the the protocols of the present study.

### Human tissues

Fifteen (15) fresh NSCLC tissues and the matched adjacent normal epithelial tissues were from primary written-informed consent NSCLC patients (stage-III-IV) in authors' institutions. Tissue slides were tested via immunohistochemistry (IHC) staining using the described protocols 32.

### YME1L shRNA or overexpression

The lentivirus encoding YME1L shRNA ((5'-GATCCCCGTGGCAGAGGAATTCATATTTCAAGAGAATATGAGTTCCTCTGCCACTTTTTGGAAA-3') or *YME1L* cDNA were provided by Dr. Cao 28 and were added to the described cells. After 48h, cells were maintained under puromycin-containing medium for another four passages. YME1L silencing or overexpression was verified at mRNA and protein levels. For xenograft studies, YME1L shRNA sequence/shC sequence was inserted into an AAV (adeno-associated virus) vector 28, 29. The construct and the AAV envelope plasmids were co-transfected to HEK-293 cells to generate shRNA-expressing AAV.

### YME1L knockout (KO) by Cas9-sgRNA (single guide RNA) method

Cells were first transduced with a dCas9-expressing construct 33 to generate dCas9-expressing stable cells 34. Next, the lentivirus with the sgRNA-CRISPR/dCas-9-YME1L-KO construct (Target DNA Sequence: *ATGATGTCGATACAAGCAAG*, PAM Sequence: *AGG*, provided by Dr. Cao 28) was added to dCas9-expressing NSCLC cells, and stable cells established following selection and YME1L KO screening. The lenti-CRISPR/dCas-9 vector encoding the non-sense sgRNA (“Cas9C”) was transduced to the control cells.

### Thiobarbituric acid reactive substance (TBAR) assaying of lipid peroxidation

Tissue lysates, at 20 proteins per sample, were measured using a commercial TBAR kit (Cayman Chemical, MI) specifically quantifying lipid peroxidation and malondialdehyde (MDA) contents colorimetrically. TBAR intensity was examined at 555 nm with the reference of 590 nm.

### Other cell functional assays and gene/protein expression/interaction detection

NSCLC cells/epithelial cells with the designated YEM1L genetic treatment were seeded at optimized confluence and cultivated. CCK-8 cell viability, colony formation, the nuclear EdU/DAPI staining assay of cell proliferation, the caspase-3 activity assay, cell apoptosis detection by nuclear TUNEL/Hoechst 33342 staining, *in vitro* cell migration “Transwell” assays, JC-1 staining of mitochondrial depolarization were described in detail in our previous studies 30, 31. Trypan blue staining of cell death and ssDNA (single strand DNA) ELISA (Merck, Shanghai, China) were described in a previous study 35, with the Histone-bound DNA ELISA assays described in another study 36. Quantitative real time-PCR (qPCR), Western blotting and co-immunoprecipitation (Co-IP) was described early as well 30, 31. CellROX staining of ROS content, tissue/cellular ATP contents and the mitochondrial complex I activity were measured using the described protocols 29, 37. Figure **S1** listed the uncropped blotting images.

### Constitutively-active mutant Akt1 (caAkt1)

The caAkt1 (S473D)-expressing adenovirus (from Dr. Li 38, 39) was added to cultured NSCLC cells for 48h. With selection the single stable cells were formed, and caAkt1 expression always verified.

### Animal studies

The nude mice were purchased from the animal center described 30, 31. The pNSCLC-1 cells (3 × 10 ^6^ cells of each mouse) were subcutaneously (*s.c.*) injected to mice's right flanks. After 21 days the pNSCLC-1 xenograft tumors were formed, with tumors close to 100 mm^3^. The pNSCLC-1 xenograft mice were intratumorally injected with the described AAV (2.5 μL virus per xenograft, 0.85×10 ^9^ PFU). Tumor xenografts were measured formulas described 30, 31. All animal experiments were approved by Nanchang University's IACUC and Animal Ethics Board.

### Statistical analysis

The detailed procedures of statistical analyses were reported in our previous studies 30, 31. All *in vitro* experiments were repeated five times. Error bars were mean ± standard deviation (SD).

## Results

### YME1L expression is elevated in NSCLC

**Figure 1:**
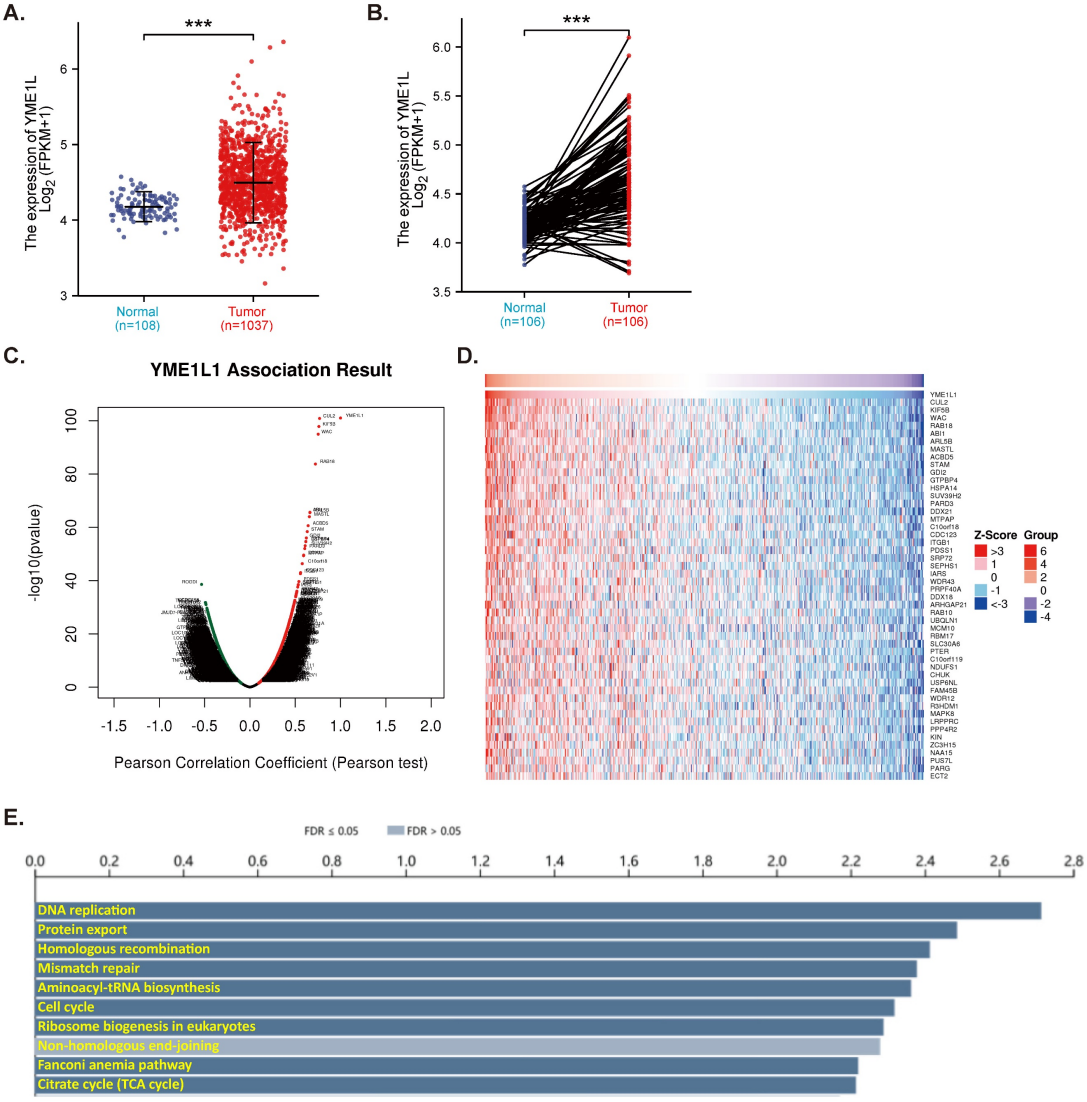
** YME1L expression is elevated in NSCLC.** TCGA-LUAD cohort showed *YME1L* expression (RNA-Seq) in 1037 NSCLC tissues (“Tumor”) and 108 normal lung tissues (“Normal” **(A)**. TCGA-LUAD cohort showed *YME1L* expression in 106 NSCLC tissues (“Tumor”) and matched 106 adjacent normal tissues (“Normal”) **(B)**. LinkedOmics functional assays demonstrated the YME1L-co-expressed genes **(C)**. Top 50 co-expressed genes positively correlated with *YME1L* **(D)** and top the enriched pathways (through KEGG, **E**) were shown. *** ***P*** < 0.001 **(A and B)**.

TCGA-LUAD database reveals that expression of *YME1L* is significantly elevated in NSCLC tissues (“Tumor”, Figure **1A**). The relatively low *YME1L* expression was detected in lung tissues (“Normal”, Figure **1A**). In NSCLC tumor tissues *YME1L* transcripts' number is robustly higher than its number in the adjacent normal tissues (Figure **1B**). The mRNA sequencing data from a total of 515 NSCLC-LUAD patients in TCGA were analyzed using the LinkedOmics functional module. Figure **1C**, the volcano plot, shows red dot genes that were positively correlated with *YME1L*, whereas green dot genes were negatively correlated with YME1L (false discovery rate/FDR < 0.01). The top fifty genes positively correlating with *YME1L* expression were presented in a heat map (Figure **1D**). Significant KEGG term annotation by overrepresentation enrichment analysis (ORA) showed the top ten pathways enriched by *YME1L*-co-expressing genes (Figure **1E**). Many of these pathways are vital for cancer progression, including DNA replication, mismatch repair, cell cycle progression and citrate cycle (TCA cycle) (Figure **1E**). These bioinformatics studies show that overexpressed YME1L might exert a tumorigenic role in NSCLC.

### YME1L expression is elevated in local NSCLC

**Figure 2:**
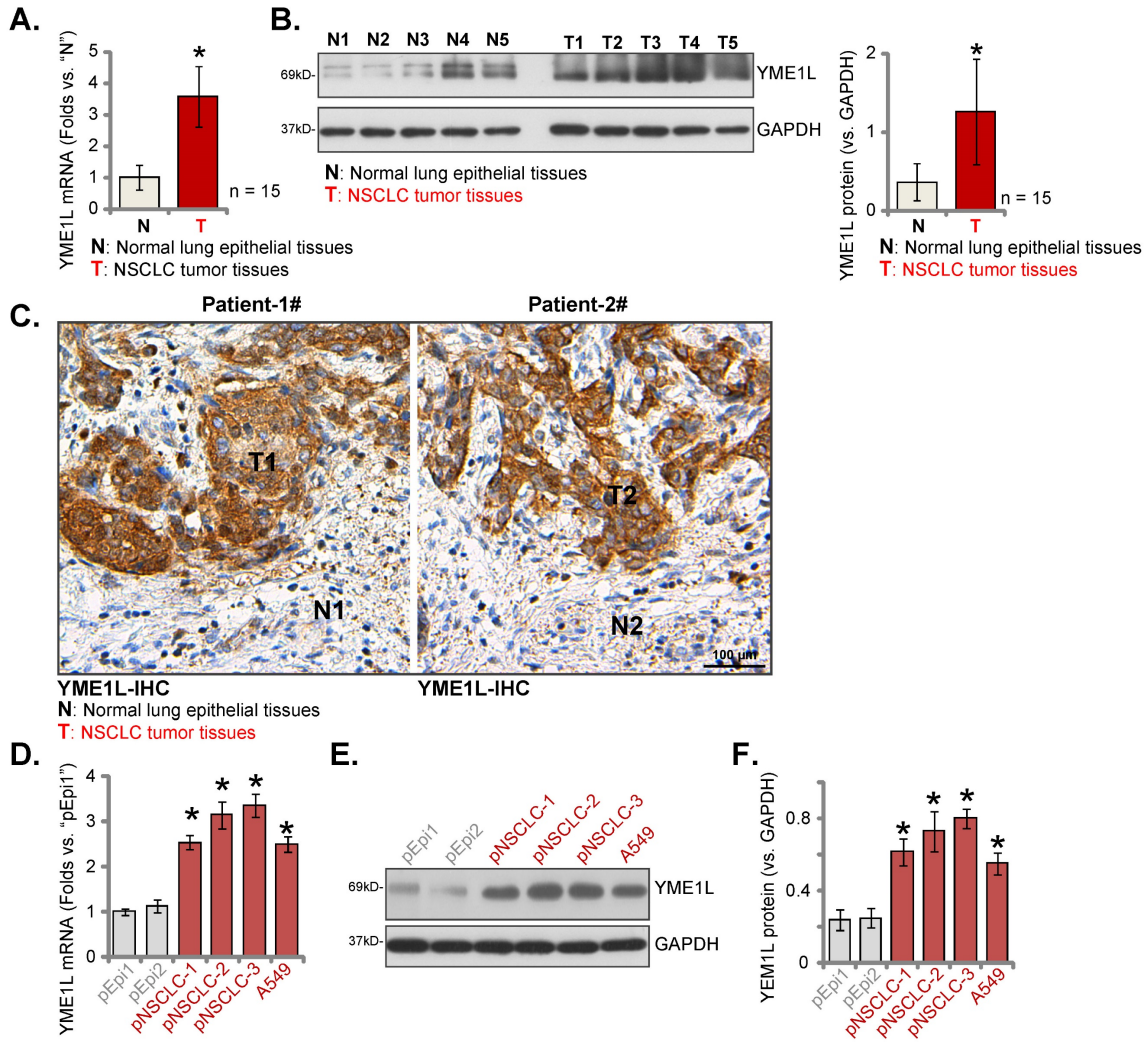
** YME1L expression is elevated in local NSCLC.** YME1L expression in the descried NSCLC tumor tissues (“T”) and matched adjacent normal lung epithelial tissues (“N”) of fifteen (n = 15) primary local NSCLC patients was examined **(A and B)**; YME1L IHC images in the described tissue slides were shown **(C)**. YME1L expression in the descried NSCLC cells/epithelial cells was measured **(D-F)**. * ***P*** < 0.05 versus “N” tissues or “pEpi1” cells. Scale bar = 100 μm.

YME1L expression was measured in local surgery-resection NSCLC tissues. NSCLC tissues (“T”) and matched adjacent normal tissues (“N”) were obtained from fifteen (n = 15) primary NSCLC patients (LUAD, stage III-IV). Analyzing tissue lysates confirmed that *YME1L* mRNA levels in NSCLC tumor tissues were dramatically higher than those in normal tissues (Figure **2A**). Moreover, increased protein expression of YME1L was observed in NSCLC tissues of five patients (“T1” to “T5”) (Figure **2B**). Quantified results combining YME1L protein blotting data of the 15 sets of tissues demonstrated that the protein expression of YME1L was significantly elevated in NSCLC tissues (Figure **2B**, the right panel). The tissue IHC images further supported the protein upregulation of YME1L in NSCLC tissues (“T1” and “T2”) of “Patient-1# and Patient-2#” (Figure **2C**). The protein expression of YME1L in normal lung epithelial tissues was low (Figure **2C**).

In the primary pNSCLC-1, pNSCLC-2 and pNSCLC-3 cells 30, 31 and the immortalized A549 cells, *YME1L* mRNA expression was higher than its expression in “pEpi1” and “pEpi2” primary lung epithelial cells (Figure **2D**). The protein expression of YME1L was upregulated in the NSCLC cells (Figure **2E** and **F**), and low expression detected in the lung epithelial cells (Figure **2E** and **F**). Thus, elevated YME1L expression is detected in local NSCLC tissues and NSCLC cells.

### YME1L depletion induces robust anti-NSCLC cell activity

**Figure 3:**
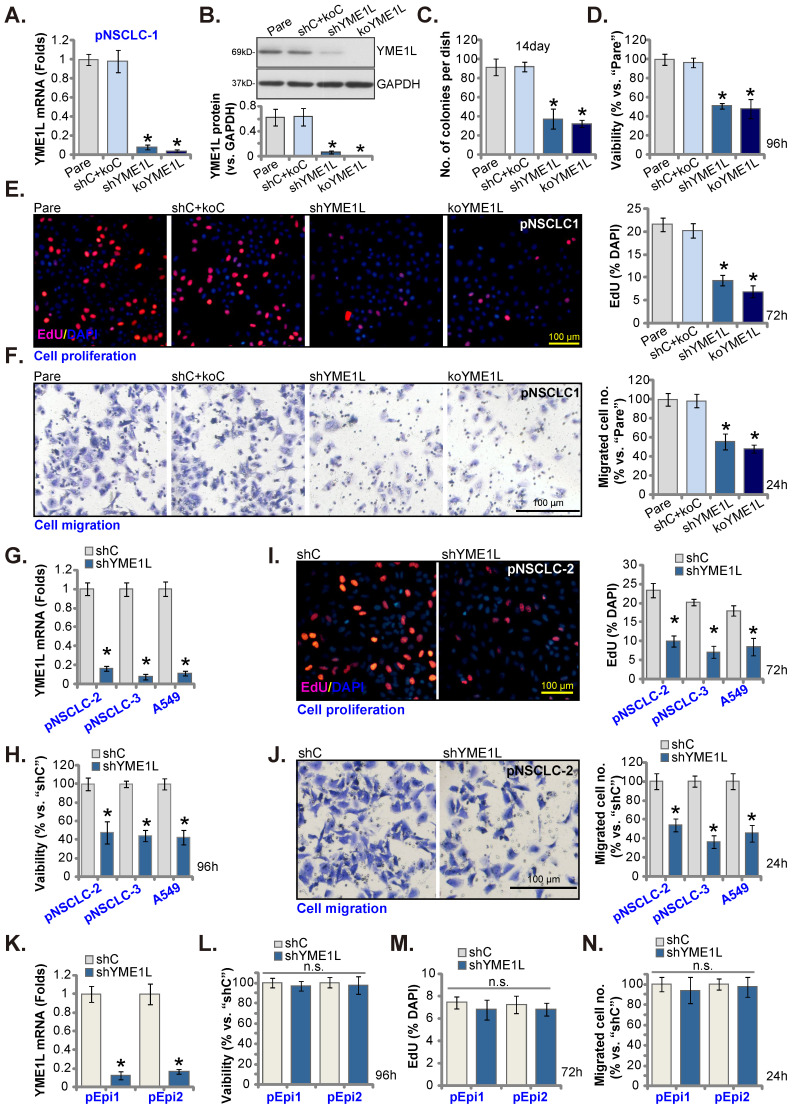
** YME1L depletion induces robust anti-NSCLC cell activity.** YME1Lexpression in pNSCLC-1cells with the described genetic modification of YME1L or with control treatment was shown **(A and B)**, and GAPDH tested as the internal control. Cells were cultivated, clonogenicity **(C)**, CCK-8 viability **(D)**, EdU incorporation **(E)** and cell migration **(F)** were measured. pNSCLC-2/3 cells, A549 cells, or pEpi1/2 epithelial cells, stably expressing YME1L shRNA (“shYME1L”) or control shRNA (“shC”) were formed, and *YME1L* mRNA expression examined **(G and K)**. Cells were cultivated, CCK-8 viability **(H and L)**, nuclear EdU incorporation **(I and M)** and migration **(J and N)** were measured, with results quantified. “Pare” stands for the parental control cells (same for all Figures). * ***P*** < 0.05 versus “Pare”/ “shC”. “n.s.” stands for ***P*** > 0.05 (same for all Figures). Scale bar=100 μm.

Aiming to knockdown YME1L, the YME1L shRNA-encoding lentivirus was added to pNSCLC-1 primary cells 30, 31, and stable cells, “shYME1L” cells, formed following selection. Alternatively, the CRISPR/dCas9-YME1L-KO construct-expressing lentivirus was added to dCas9-expressing pNSCLC-1 cells, and single stable “koYME1L” cells formed following selection and *YME1L* KO verification. When compared to pNSCLC-1 cells with the non-sense scramble control shRNA and the CRISPR/dCas9-KO control construct (“shC+koC”), *YME1L* mRNA (Figure **3A**) and protein (Figure **3B**) were substantially decreased in shYME1L and koYME1L pNSCLC-1 cells. As a result, cell colony formation ability was robustly inhibited (Figure **3C**). YME1L shRNA/KO also potently decreased viability in pNSCLC-1 cells (Figure **3D**). Further studies showed that depletion of YME1L significantly inhibited pNSCLC-1 cell proliferation by decreasing the percentage of EdU nuclei (Figure **3E**). Furthermore, pNSCLC-1 cell motility was suppressed after YME1L depletion. The *in vitro* cell migration (Figure **3F**) was largely inhibited in shYME1L and koYME1L cells. Therefore, depletion of YME1L inhibited pNSCLC-1 cell survival, proliferation and motility.

Next, to pNSCLC-2/3 cells and immortalized A549 cells, the lentivirus with YME1L shRNA was added and stable “shYME1L” cells were formed following selection. Expression of *YME1L* mRNA was indeed robustly decreased in the shYME1L NSCLC cells (Figure **3G**). Silence of YME1L in the NSCLC cells decreased viability (Figure **3H**), arrested cell proliferation (Figure **3I**) and hindered *in vitro* cell migration (Figure **3J**).

The pEpi1 and pEpi2 primary epithelial cells 30, 31 were also transduced with the lentiviral YME1L shRNA (“shYME1L”). The latter led to dramatic YME1L silencing (Figure **3K**). Yet YME1L shRNA failed to significantly inhibit viability (Figure **3L**), EdU incorporation (Figure **3M**) and *in vitro* cell migration (Figure **3N**) in pEpi1/2 epithelial cells.

### YME1L depletion provokes NSCLC cell apoptosis

**Figure 4:**
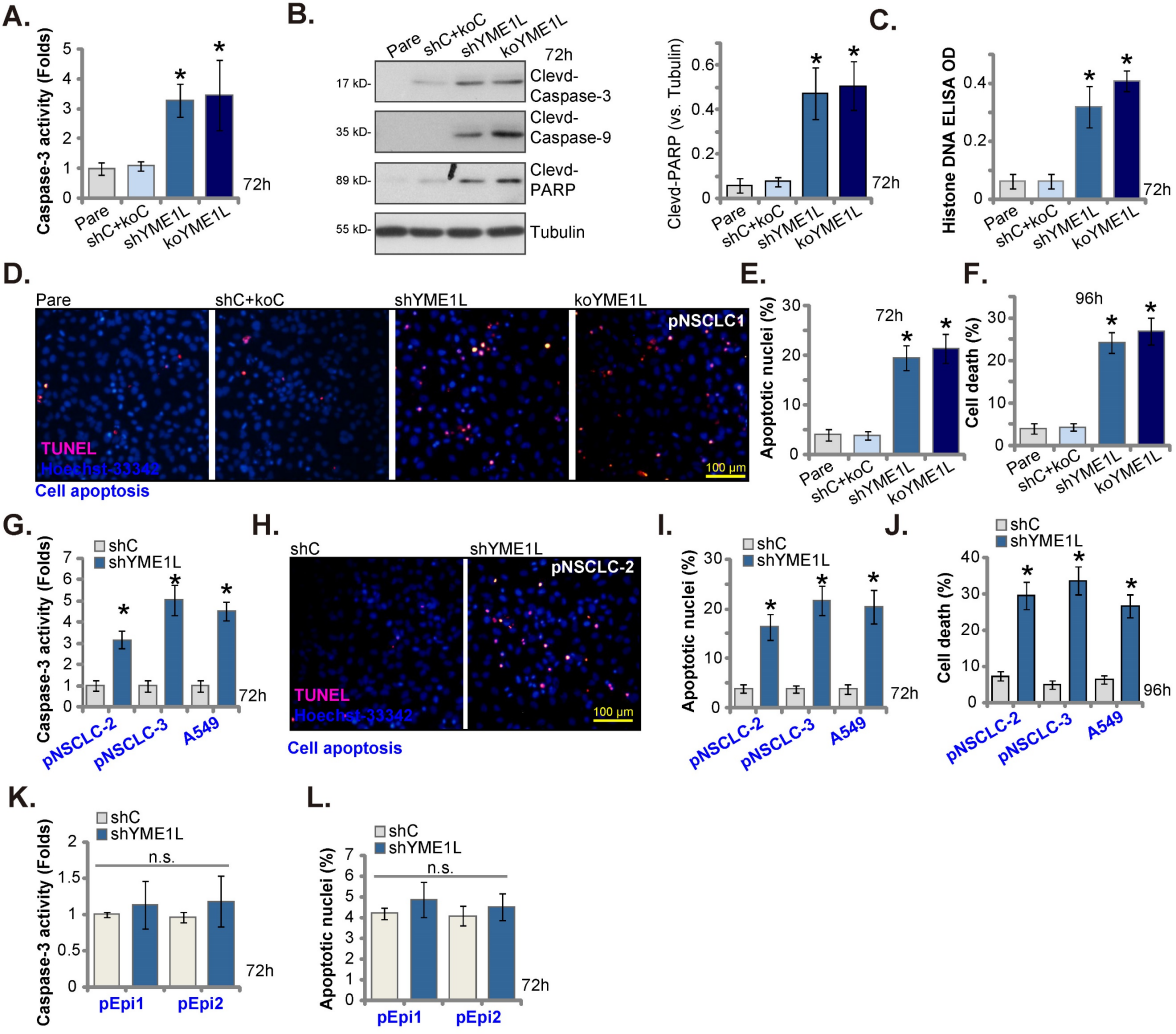
** YME1L depletion provokes NSCLC cell apoptosis.** pNSCLC-1cells with the described genetic modification of YME1L or with control treatment were cultivated, the caspase-3 activity was tested **(A)**, and listed proteins measured **(B)**; The histone-DNA contents were tested **(C)**. Cell apoptosis was tested via the nuclear TUNEL staining assay **(D and E)**, and cell death measured by the Trypan blue staining assay **(F)**. pNSCLC-2/3 cells, A549 cells, or pEpi1/2 epithelial cells, stably expressing YME1L shRNA (“shYME1L”) or control shRNA (“shC”) were cultured, the caspase-3 activity was measured **(G and K)**, with cell apoptosis measured via the TUNEL staining assays **(H**, **I and L)**. Cell death was measured as well **(J)**. * ***P*** < 0.05 versus “Pare”/ “shC” cells. Scale bar=100 μm.

We tested whether YME1L depletion could induce apoptosis activation. As shown, in shYME1L and koYME1L pNSCLC-1 cells (see Figure **3**), Caspase-3 activity was significantly increased when compared to that in shC+koC control cells (Figure **4A**). Moreover, in pNSCLC-1 cells YME1L shRNA/KO caused Caspase-3, PARP1 and Caspase-9 cleavages (Figure **4B**). The contents of Histone-bound DNA were increased in YME1L-silenced/-KO pNSCLC-1 cells (Figure **4C**). Importantly, following YME1L shRNA/KO, the ratio of nuclei with TUNEL staining was significantly increased, supporting apoptosis induction (Figure **4D** and **E**). Increased cell death was detected in shYME1L and koYME1L pNSCLC-1 cells and Trypan blue-positive staining was significantly increased (Figure **4F**).

In pNSCLC-2/3 cells and A549 cells, YME1L shRNA-induced silence of YME1L augmented Caspase-3 activity (Figure **4G**) and increased TUNEL nuclei ratio (Figure **4H** and **I**). Moreover, YME1L silencing provoked cell death and increased Trypan blue staining in the NSCLC cells (Figure **4J**). Whereas in pEpi1 and pEpi2 lung epithelial cells, YME1L knockdown by YME1L shRNA (see Figure **3**) failed to provoke apoptosis (Figure **4K** and **L**).

### NSCLC cell mitochondrial functions are impaired after YME1L depletion

**Figure 5:**
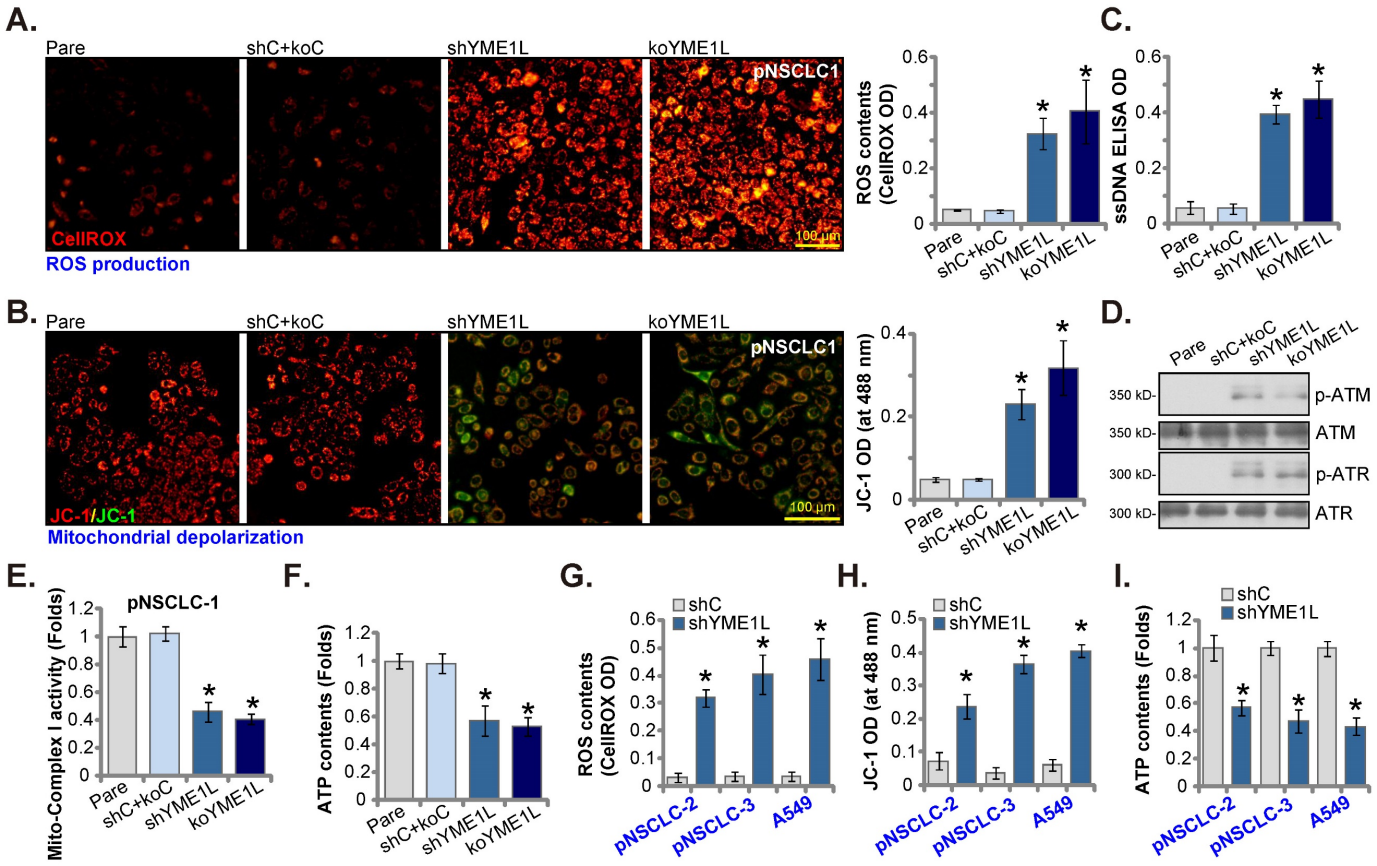
** NSCLC cell mitochondrial functions are impaired after YME1L depletion.** pNSCLC-1cells with the described genetic modification of YME1L or with control treatment were cultivated for 48h, CellROX intensity **(A)**, depolarization of mitochondria (JC-1 monomer intensity, **(B)**, DNA breaks (ssDNA contents, **(C)** and ATM/ATR phosphorylation and expression **(D)** were tested. The activity of mitochondrial complex I **(E)** and intracellular ATP contents **(F)** were examined as well. pNSCLC-2/3 cells, A549 cells, or pEpi1/2 epithelial cells, stably expressing YME1L shRNA (“shYME1L”) or control shRNA (“shC”) were cultured for 48h, CellROX intensity **(G)**, JC-1 monomer intensity **(H)** and ATP levels **(I)** were measured. * ***P*** < 0.05 versus “Pare”/ “shC” cells. Scale bar=100 μm.

YME1L locates in the inner mitochondrial membrane 20-24. It maintains mitochondrial morphology and is vital for mitochondrial function and plasticity 22, 25, 26. We therefore analyzed whether mitochondrial functions were impaired in YME1L-depleted NSCLC cells. As shown, the CellROX red fluorescence intensity was substantially increased in shYME1L and koYME1L pNSCLC-1 cells, supporting ROS accumulation (Figure **5A**). In addition, YME1L shRNA/KO resulted in depolarization of mitochondria in pNSCLC-1 cells, causing JC-1 red fluorescence conversion to green JC-1 monomers (Figure **5B**). Furthermore, ssDNA accumulation and increased DNA breaks were detected in YME1L-depleted pNSCLC-1 cells (Figure **5C**). Increased ATM and ATR phosphorylation further supported DNA damage in YME1L-depleted cells (Figure **5D**). The activity of mitochondrial complex I was substantially decreased in shYME1L and koYME1L pNSCLC-1 cells (Figure **5E**). Consequently, the ATP contents were decreased (Figure **5F**).

In pNSCLC-2/3 cells and A549 cells, YME1L shRNA also induced ROS production and increased the CellROX red fluorescence intensity (Figure **5G**). Moreover, depolarization of mitochondria, reflected by green monomer JC-1 accumulation, was detected in YME1L-silenced primary and A549 NSCLC cells (Figure **5H**). In addition, ATP depletion was detected in the NSCLC cells with YME1L silencing (Figure **5I**). Thus, YME1L depletion impaired mitochondrial functions in NSCLC cells.

### Ectopic YME1L overexpression further promotes NSCLC cell growth

**Figure 6:**
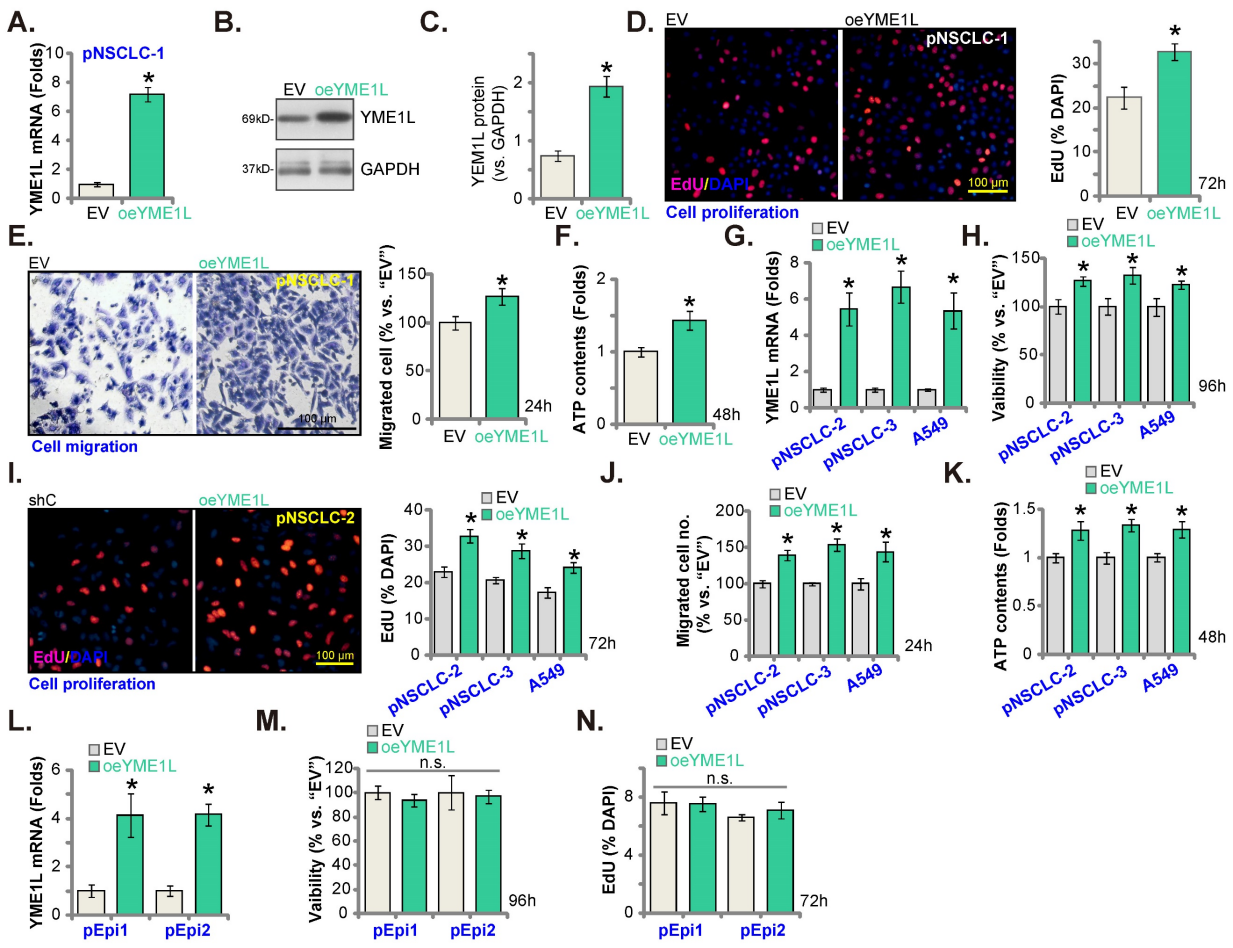
** Ectopic YME1L overexpression further promotes NSCLC cell growth.** pNSCLC-1/2/3 cells **(A-K)**, A549 cells **(G-K)** or pEpi1/2 epithelial cells **(L-N)**, stably expressing the lentivirus-packed YME1L-expressing construct (“oeYME1L”) or the vector (“EV”) were formed, mRNA and protein expression of *YME1L* was measured** (A**, **B**, **C**, **G and L)**; After further cell culturing, CCK-8viability **(H and M)**, nuclear EdU incorporation **(D**, **I and N)** and *in vitro* migration **(E and J)** were examined, with the cellular ATP contents measured **(F and K)**. * ***P*** < 0.05 versus “EV” cells. Scale bar=100 μm.

The lentivirus-packed YME1L-expressing construct was stably transduced to pNSCLC-1 cells, establishing YME1L-ovexpressed cells (“oeYME1L”). Comparing to the empty vector (“EV”)-expressing pNSCLC-1, expression of *YME1L* mRNA (Figure **6A**) and protein (Figure **6B** and **C**) was substantially elevated in the oeYME1L pNSCLC-1 cells. YME1L overexpression potentiated pNSCLC-1 cell proliferation and augmented nuclear EdU incorporation (Figure **6D**). Moreover, the *in vitro* cell migration (Figure **6E**) was augmented and ATP content (Figure **6F**) was increased in oeYME1L cells.

The lentivirus-packed YME1L-expressing construct was also transduced to pNSCLC-2/pNSCLC-3 and A549 cells. Thereafter YME1L-ovexpressed NSCLC cells (“oeYME1L”) were formed, showing significantly-elevated *YME1L* mRNA expression (Figure **6G**). With overexpression of YME1L, cell viability (Figure **6H**) and proliferation (Figure **6I**) were augmented in the NSCLC cells. Moreover, the *in vitro* migration was accelerated (Figure **6J**), and the cellular ATP content increased in oeYME1L NSCLC cells (Figure **6K**). To lung epithelial cells (pEpi1 and pEpi2), the lentivirus-packed YME1L-expressing construct was transduced and YME1L-ovexpressed stable epithelial cells (“oeYME1L”) were formed. *YME1L* mRNA expression was elevated in oeYME1L lung epithelial cells (Figure **6L**). YME1L overexpression however failed to increase viability (Figure **6M**) and nuclear EdU incorporation (Figure **6N**) in the lung epithelial cells.

### YME1L depletion in NSCLC cells disrupts mTOR complex assembling and Akt-mTOR activation

**Figure 7:**
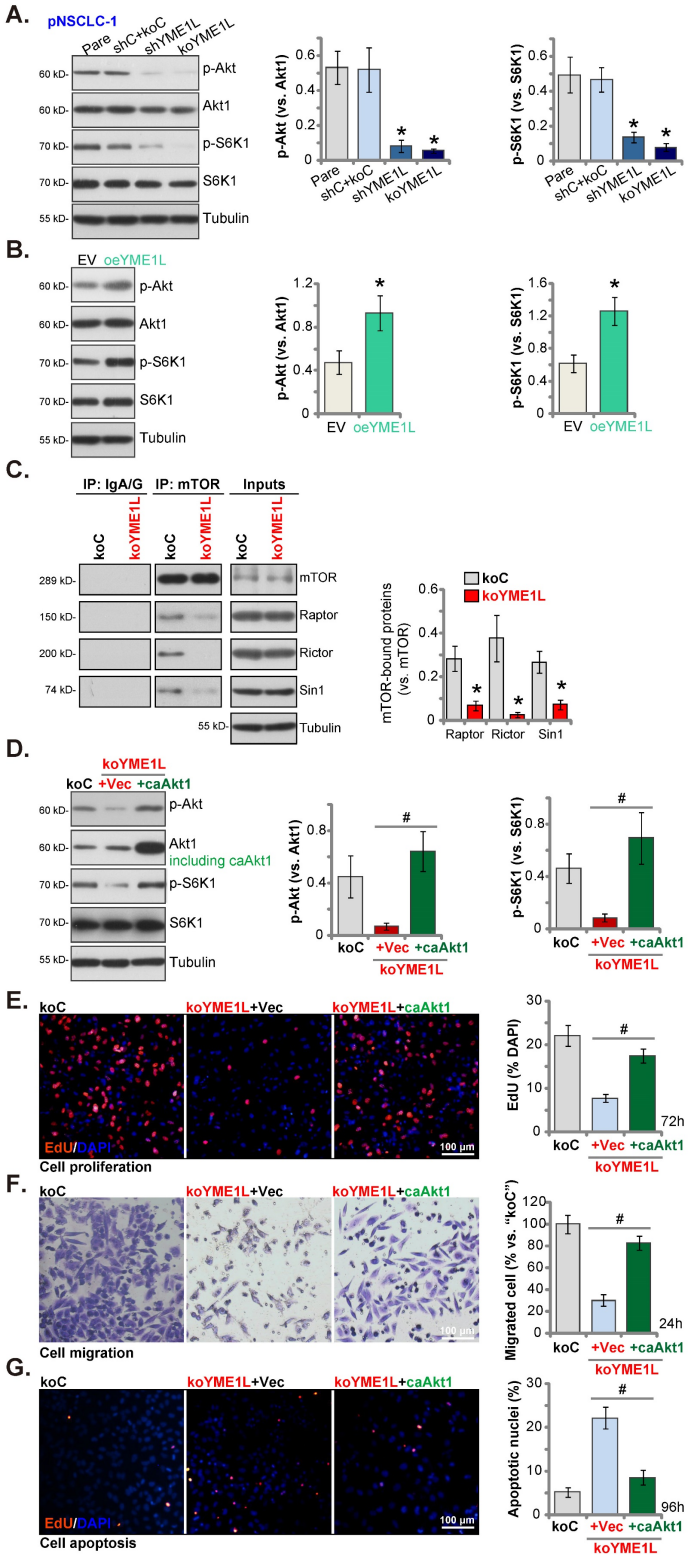
** YME1L depletion in NSCLC cells disrupts mTOR complex assembling and Akt-mTOR activation.** Listed proteins in pNSCLC-1cells with the described genetic modification of YME1L or with control treatment were tested **(A and B)**. The mTOR-immunoprecipitated proteins (Raptor, Rictor and mSin1) were measured by Co-IP assays, with expression of the described proteins measured in “Inputs” **(C)**. The koYME1L pNSCLC-1 cells were further stably transduced with caAkt1 (S473D) or the vector (“Vec”), with the listed proteins tested **(D)**; After further culturing nuclear EdU incorporation **(E)**, *in vitro* migration **(F)** and apoptosis (TUNEL-nuclei staining, **(G)** were tested. * ***P*** < 0.05 versus “shC+Cas9-C”/“EV” cells. **^#^ *P*** < 0.05. Scale bar=100 μm.

The mitochondrial function is required for the activation of Akt-mTOR and other pro-cancerous oncogenic cascades in NSCLC 40, 41. In pNSCLC-1 cells, shRNA-induced silence of YME1L (“shYME1L”) or dCas9/sgRNA-mediated KO of YME1L (“koYME1L”) remarkably decreased phosphorylation of Akt (Ser-473) and S6K1 (Figure **7A**). Total Akt1 and S6K1 were unchanged in YME1L-silenced/-KO pNSCLC-1 cells (Figure **7A**). Contrarily, in YME1L-overexpressing pNSCLC-1 cells (“oeYME1L”) Akt (Ser-473) and S6K1 phosphorylation were augmented (Figure **7B**).

There are two mTOR complexes, mTORC1 and mTORC2 42, 43. mTORC1, a complex including mTOR, mLST8, Raptor, and several others, is responsible for phosphorylating S6K1 and 4E-BP1 42, 43. mTORC2 is composed of mTOR, mSIN1, Rictor and mLST8 44-46, and phosphorylates Akt (at Ser-473) 47, 48. Both mTOR complexes are important for NSCLC tumorigenesis and progression 30, 39, 49, 50. Here the co-immunoprecipitation assay results showed that the assembles mTORC1 (association of mTOR-Raptor) and mTORC2 (association of mTOR-Rictor-mSin1) were disrupted in YME1L-KO pNSCLC-1 cells (Figure 7C). mTOR, Raptor, mSin1 and Rictor protein expression however unchanged with YME1L KO (Figure 7C, “Inputs”). Thus, disruption of the assembling of mTORC1/2 could be the primary mechanism of Akt-mTOR inactivation by YME1L depletion in NSCLC cells.

Next a caAkt1 (S473D) construct 30, 31 was stably transduced to koYME1L pNSCLC-1 cells (Figure **7D**) and completely restored Akt (Ser-473) and S6K1 phosphorylation in the YME1L KO cells (Figure **7D**). Significantly YME1L KO-induced proliferation (EdU incorporation) suppression (Figure **7E**), migration inhibition (Figure **7F**) and apoptosis (TUNEL nuclei increasing, Figure **7G**) in pNSCLC-1 cells were ameliorated by caAkt1.

### Silence of YME1L inhibits NSCLC xenograft growth

**Figure 8:**
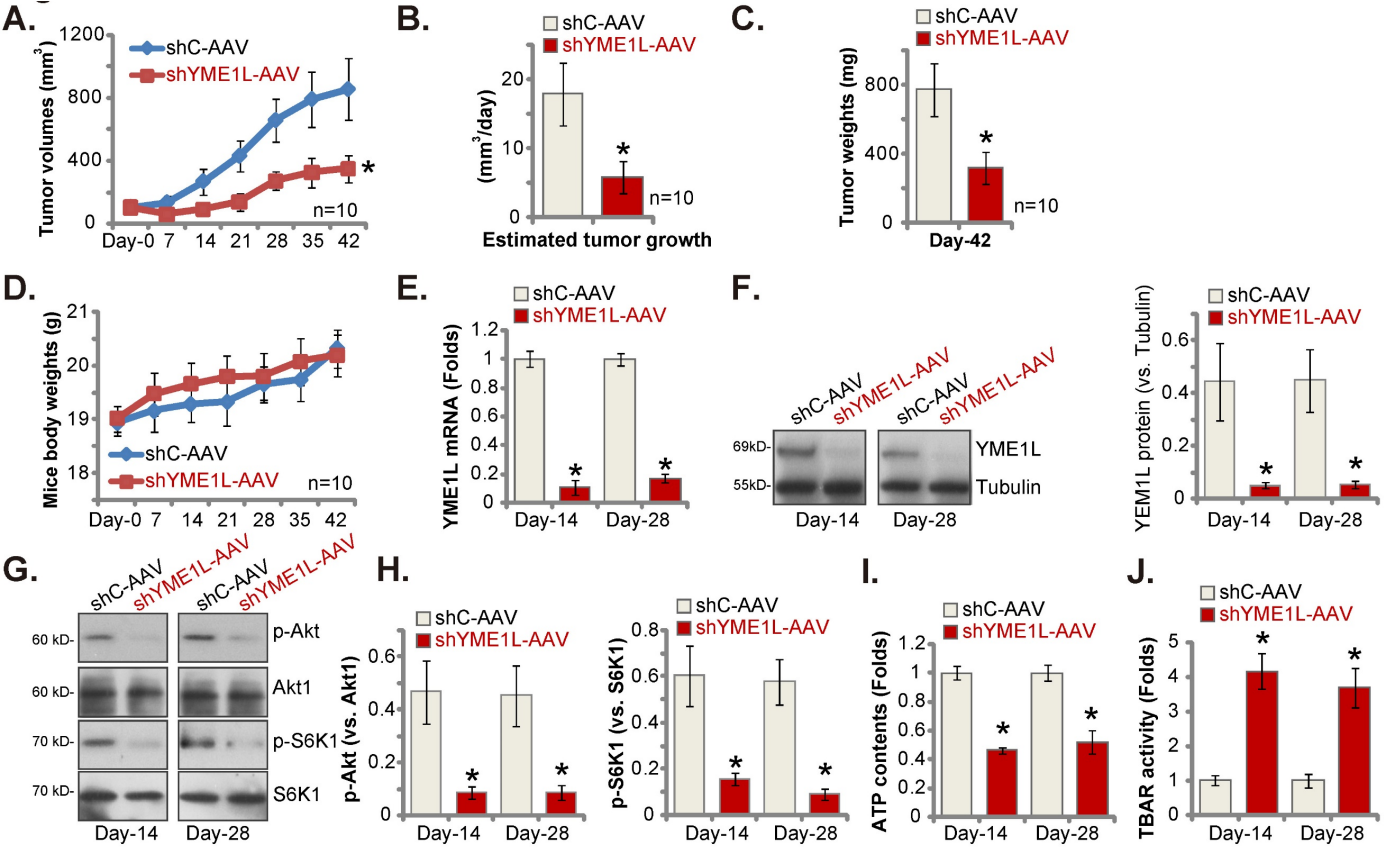
** Silence of YME1L inhibits NSCLC xenograft growth.** ThepNSCLC-1 xenograft nude mice were injected with the YME1L shRNA AAV (“shYME1L-AAV”) or control shRNA AAV (“shC-AAV”). The virus was intratumorally injected every 48h for a total of five rounds. The weekly tumor volumes **(A)** and mice body weights **(D)** were weekly recorded. The estimated daily pNSCLC-1 xenograft growth was shown **(B)**. At Day-42, pNSCLC-1 xenografts were carefully isolated and individually weighted **(C)**. Listed mRNAs and proteins in the described pNSCLC-1 xenografts were measured **(E-H)**, with ATP contents **(I)** and TBAR **(J)** activity tested as well. * ***P*** < 0.05 “shC-AAV” group. Scale bar=100 μm.

Lastly, pNSCLC-1 cells were subcutaneously (*s.c.*) injected to nude mice. The pNSCLC-1 xenograft tumors were formed after 21 days (“Day-0”). The YME1L shRNA-expressing AAV (“shYME1L-AAV”) were then intratumorally injected to the nude mice. Whereas in control mice the control shRNA AAV (“shC-AAV”) was injected. Virus injection was carried every 48h for five rounds. shYME1L-AAV injection robustly hindered pNSCLC-1 xenograft growth (Figure **8A**). The estimated daily pNSCLC-1 xenograft growth, expressed as mm^3^ per day, was calculated 30, 31. The results again showed that shYME1L-AAV treatment suppressed pNSCLC-1 xenograft growth (Figure **8B**). All the pNSCLC-1 xenografts were isolated carefully at Day-42 and individually weighted. The shYME1L-AAV-injected pNSCLC-1 xenografts injection were substantially lighter than the shC-AAV xenografts (Figure **8C**). When comparing the animal body weights, we failed to detect any significant difference between the two groups (Figure **8D**).

Signaling changes in the pNSCLC-1 xenografts were examined. Specifically at Day-14 and Day-28, one pNSCLC-1 xenograft from the shYME1L-AAV group and the shC-AAV group was carefully isolated. Part of the fresh tumor xenografts were cut into five pieces and signalings were tested. YME1L expression was remarkably decreased in shYME1L-AAV pNSCLC-1 xenograft tissues (Figure** 8E** and **F**), where Akt (Ser-473) and S6K1 phosphorylation was robustly decreased (Figure **8G** and **H**). Total Akt1 and S6K1 was again unaffected by shYME1L-AAV (Figure **8G** and **H**). Moreover, ATP reduction was detected in shYME1L-AAV xenografts (Figure **8I**). Lipid peroxidation, or increased TBAR activity, was detected in YME1L-silenced pNSCLC-1 xenograft tissues (Figure **8J**).

## Discussion

Srinivasainagendra *et al.*, reported that in human colorectal cancer YME1L could be frequently mutated, and its mutation also occurring in other human cancers to a less degree 51. YME1L inhibition led to significant death of cancer cells 23, 52. Silence of YME1L caused accumulation of Ndufb6, ND1, and Cox4, thereby suppressing cell proliferation 23.

Recent studies have proposed a possible tumorigenic role of YME1L. Liu *et al.,* have shown that overexpressed YME1L is important for orthotopic glioma xenograft growth in mice 28. The same group further reported that TIMM44, another mitochondrial protein, promoted glioma cell growth possibly by increasing YME1L transcription and expression 29. Liao *et al.,* have implied that YME1L could be a promising biomarker for diagnosis and prognosis prediction in ovarian cancer 53. YME1L is upregulated in ovarian cancer and is associated with worse overall survival 53. Moreover, YME1L and it co-expressing genes are enriched in immune-related signaling pathways, supporting a possible inhibitory role of YEM1L in cancer immunotherapy 53. Kakehashi *et al.,* reported that expression of YME1L, together with other cytoskeletal proteins involved in endoplasmic reticulum stresses and mitochondrial dysfunctions, are overexpressed in HCV-associated hepatocellular carcinomas (HCC) 54.

Increased mitochondrial respiration and ATP generation are extremely important for NSCLC tumorigenesis and progression. Here we found that the mitochondrial protein YME1L exerted tumorigenic activity in NSCLC. TCGA database and local human tissues/cells results demonstrated that YME1L expression is elevated in NSCLC tissues and cells. YME1L shRNA or KO potently suppressed NSCLC cell viability, proliferation and *in vitro* migration, and provoking apoptosis. In addition, YME1L depletion caused mitochondrial dysfunctions, leading to depolarization of mitochondria, oxidative injury, DNA breaks and ATP depletion in different NSCLC cells. *In vivo*, the growth of subcutaneous primary NSCLC xenografts was hindered following YME1L shRNA AAV injection in nude mice. ATP reduction and oxidative injury were observed in YME1L-depleted xenograft tissues.

Due to mutation and other genetic alterations, increased activation of PI3K-Akt-mTOR cascade is often detected in NSCLC 7, 10, 55-57. Conversely, small molecular inhibitors or genetic modifications that can inactivate this cascade have shown promising anti-NSCLC efficiency 7, 10, 55-57. PQR620, a mTOR kinase inhibitor, prevented mTORC1/2 activation and arrested NSCLC cell growth 30. ASP4132, the highly effective AMPK activator, suppressed NSCLC cell growth possibly via inhibiting Akt-mTOR signaling 31.

YME1L was recently shown to promote Akt activation 28. Here activation of Akt-mTOR was reduced after YME1L shRNA/KO in primary NSCLC cells. It was augmented after ectopic YME1L overexpression. Our results supported that YME1L should be important for the integrity of mTORC1/2 and YME1L depletion disrupted the assembling of mTORC1/2. Importantly, YME1L KO-mediated anti-NSCLC cell activities, including proliferation arrest, migration inhibition, and apoptosis, were largely ameliorated by caAkt1. Akt-mTOR inhibition was observed in YME1L-silenced NSCLC xenograft tissues. Thus, Akt-mTOR activation is important for YME1L-promoted NSCLC cell growth.

## Conclusion

The mitochondrial protein YME1L protein is overexpressed in NSCLC and exerts significant pro-tumorigenic activity possibly by supporting mitochondrial function and promoting Akt-mTOR activation.

## Supplementary Material

Supplementary figures.
