# Supplementary material for: The mitochondrial protein YME1 Like 1 is important for non-small cell lung cancer cell growth
Source: Int J Biol Sci. 2023 Mar 21;19(6):1778–90. doi: 10.7150/ijbs.82217 (PMC10092760; doi:10.7150/ijbs.82217)

Figure S1: The uncropped blotting images of the study

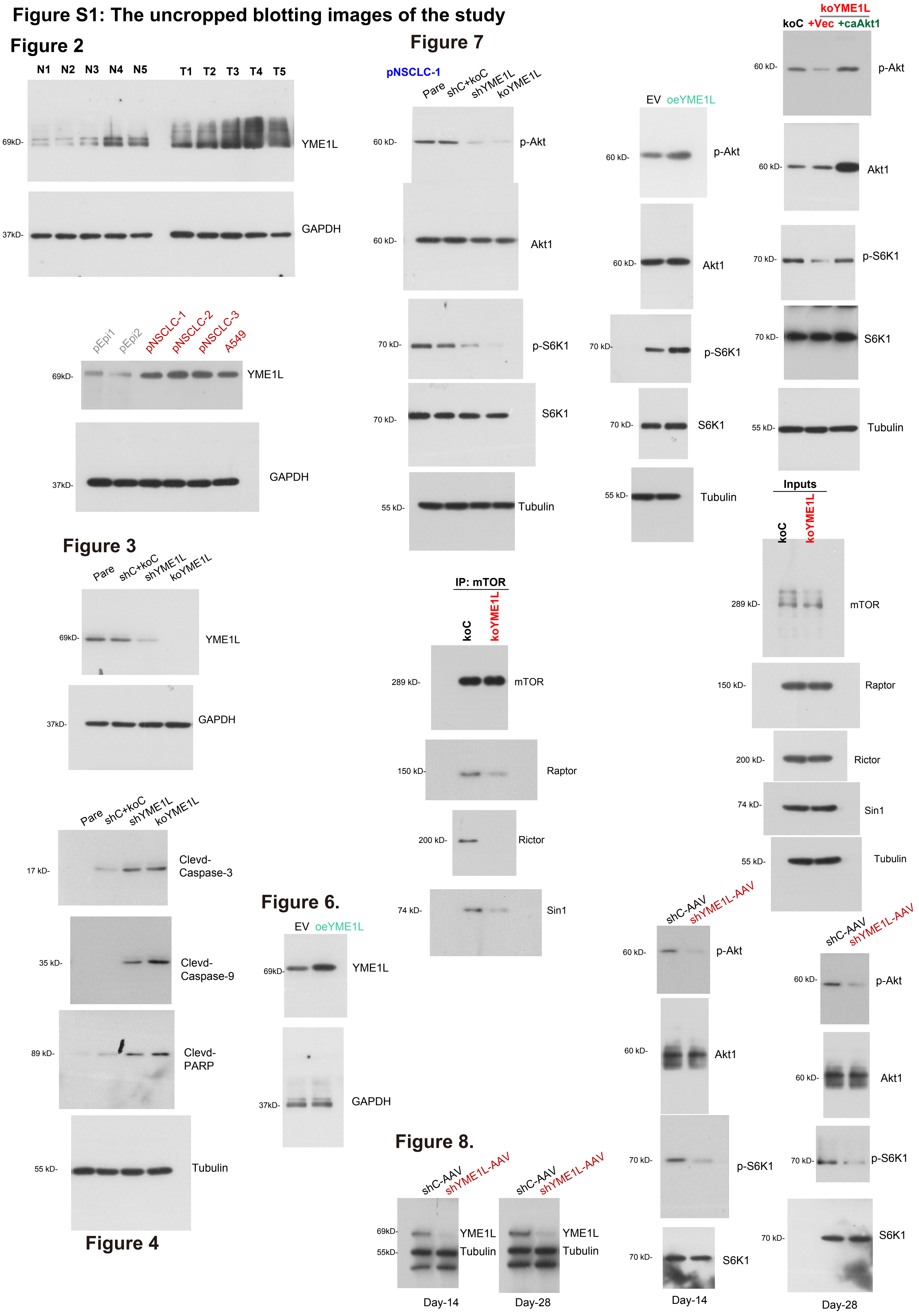

Supplement: Supplementary file 1 — Supplementary figures. [file ijbsv19p1778s1.pdf]
